# Notes and News

**Published:** 1893-06-24

**Authors:** 


### NOTES AND NEWS.

COLLECTED AND COMMUNICATED.

AT the last general court of the Radcliffe Infirmary, Oxford, the plans for a new block to extend the hospital were accepted. The committee have adhered to their intention of "walking down the garden," so to speak, and more of the invaluable free space at the back of the institution is to be encroached upon. The cost of the new block for male patients is estimated at £4,850.

THE "Memoirs of Sir Morell Mackenzie," by Mr. Haweis, are being the subject of dispute between that gentleman and the family of the late Sir Morell. In spite of their distinct objection, the book has been published, and however the matter may be settled between the disputants, the stimulation that public curiosity has received will go far towards making the book a financial success.

THE Princess Christian honoured the Sick Children's Hospital, Glasgow, with a visit, when passing through the city after her visit to Edinburgh. The hospital was gaily decorated, and H.R.H. was presented with a satin sachet containing the report and other particulars concerning the institution. She expressed herself hightly satisfied with all the arrangements. Later on the Princess visited the Nursing Institute in Bath Street, and the Royal and Western Infirmaries.

THE memorial stone of the new Halifax Infirmary was laid on Saturday last, with Masonic observances, by the Earl of Mount Edgcumbe. The occasion excited the greatest interest, many of the buildings being lavishly decorated in the central part of the town. The processions were brilliant and striking, societies of all descriptions as well as private individuals being in the show, which was also used as a means of collecting money for the hospital. A large number of Sunday school children placed a collection of £600 in purses on the stone after the ceremony. We hope to give a description of the building at a later date.

THE Skegness Branch of the Nottingham Convalescent Homes was opened on the 8th inst. We hope to give a description of the building later on.

H.R.H. THE PRINCESS MARY ADELAIDE, Duchess of Teck, and the Princess May have graciously signified their consent to lay the foundation stone of the New Homœopathic Hospital in Great Ormond Street on the 23rd inst. Mr. G. A. Cross, the Secretary-Superintendent, asks us to inform the public that the wards and out patient department, now situated in Powis Place, at the side of the old hospital, have been adequately fitted up for in and out patient work, having excellent wards and complete accommodation. In-patients are being admitted at the rate of about 500 yearly, against 800 in previous years, and out-patients at the rate of 9,000, against 10,000 in previous years. The Nursing Institute and offices have been removed to 35, Queen Square, adjoining the old hospital, and to complete the new building most probably £10,000 more will be requisite.

OF all hospital questions, none offer greater difficulties than those affecting the erection of fever hospitals, and the choice of sites for the same. The Sanitary Committee of the Commissioners of Sewers held a meeting recently to consider the question of acquiring property in Upper Thames Street, on which to erect a fever hospital. After much discussion the consideration of the question was adjourned. Whilst sympathizing with the authorities for the steady opposition which they have to encounter from those dwelling in the vicinity of every proposed site, we cannot but feel that it is imperative that these difficulties should be surmounted. It is admitted that the Asylums Board is not able to accommodate more than fifty per cent. of the fever cases in the metropolis, and to what an extent disease is unnecesarily spread in consequence, those working amongst the crowded dwellings of poorer London can and do testify.

HIS Royal Highness the Duke of York, as President, has issued an appeal on behalf of St. Mary's Hospital, accompanied by an interesting little pamphlet, to which he refers. His Royal Highness writes: "I earnestly urge upon the generosity of the public the claims of the great undertaking described in the accompanying pages—the Clarence Memorial Wing of St. Mary's Hospital. The hospital is an admirably managed institution, earnestly engaged in ministering to the sick poor of a large and densely populated district. Owing to its inadequate size all the suitable applicants for admission cannot be accommodated. Many have therefore to be sent away, while extra beds have constantly to be put up for patients so ill that, though all the regular beds are occupied, their admission is imperative. Lack of room has also compelled the authorities to lodge part of the nursing staff out of the hospital. The Clarence Memorial Wing will supply the additional accommodation thus urgently required, and, as its frontage will face Praed-street, the progress of the hospital will no longer be impeded, as it has been in the past, by an obscure situation. Unquestionably this is a most necessary work, and therefore, as President of the hospital, I strongly commend it to public support. —GEORGE, President. June, 1893."

